# Establish a visual loop-mediated isothermal amplification (LAMP) method for rapid detection avian colibacillosis

**DOI:** 10.3389/fvets.2026.1922461

**Published:** 2026-09-25

**Authors:** Anqi Ju, Hongxiang Zhang, Guoyin Wang, Rining Zhu, Siyue Wang, Chengcheng Tan, Fangyu Zhang, Chengyu Wang, Tian Wei, Dan Xiao, Pengju Zhang

**Affiliations:** 1 Jilin Academy of Agricultural Sciences (Northeast Innovation Center for Agricultural Science and Technology in China), Jilin, China; 2 College of Animal Science and Technology, Jilin Agricultural University, Jilin, China

**Keywords:** avian, *E. coli*, LAMP, rapid detection, visual

## Abstract

**Objective:**

To develop a rapid visual loop-mediated isothermal amplification (LAMP) assay for the detection of pathogenic *Escherichia coli* in poultry-associated samples.

**Methods:**

A total of 650 samples were collected from 11 large-scale poultry farms in Jilin Province, China, from which 632 bacterial isolates were recovered. Four sets of LAMP primers were designed based on conserved genomic regions identified by sequence alignment. Reaction temperature, incubation time, and Bst DNA polymerase dosage were optimized. The limit of detection, visual colorimetric performance, and cross-reactivity were evaluated, and the clinical diagnostic performance was compared with conventional PCR.

**Results:**

The optimized LAMP assay produced detectable amplification within 45 min at 63 °C, with a limit of detection of 3.6 CFU/mL. Positive reactions turned yellow-green, whereas negative reactions remained orange, enabling naked-eye discrimination. No cross-reactivity with non-target bacterial species was observed. The LAMP assay exhibited a 1,000-fold lower limit of detection than conventional PCR, required neither thermal cycling nor gel electrophoresis, and showed complete agreement with PCR results for clinical samples.

**Conclusion:**

This colorimetric LAMP assay is reliable and holds considerable promise as a rapid on-site screening tool for pathogenic *E. coli* in poultry-associated samples.

## Graphical Abstract

**Figure:**
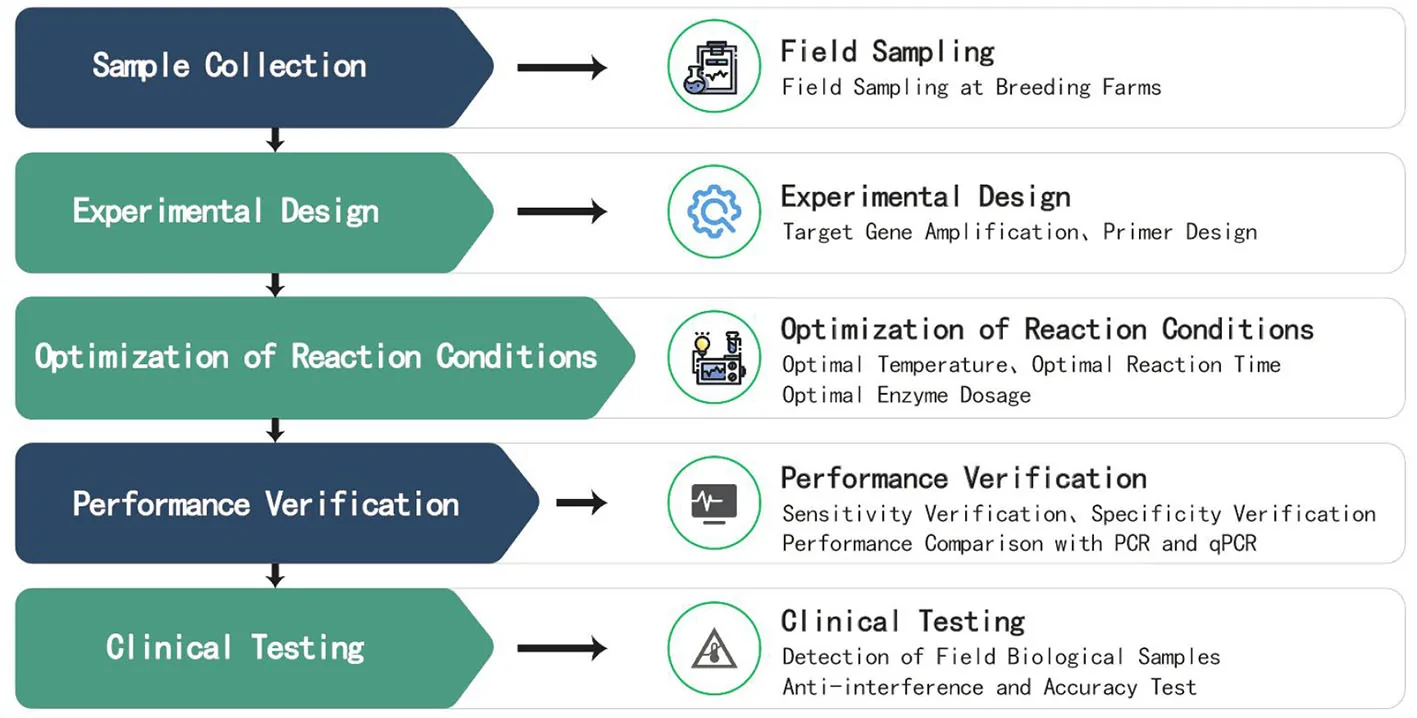

## 1 Introduction

*Avian colibacillosis* (AC) is a well-known bacterial infection linked to harmful strains of *Escherichia coli* (*E.coli)* (1). This disease manifests through various symptoms such as pericarditis, septicemia, air sacculitis, perihepatitis, salpingitis, and yolk peritonitis [1, 2](2). The high rates of occurrence and mortality associated with AC pose a serious threat to the global poultry sector (3). Traditionally, diagnosing avian *E.coli* infections involves isolating and culturing the bacteria, followed by biochemical and serotype identification (4). However, this approach is impractical for routine use due to its labor-intensive and time-consuming nature (5). Recently, several PCR techniques, including conventional PCR, nested PCR, real-time RT-PCR (RT-qPCR), and nanoparticle-assisted PCR (NanoPCR), have gained popularity for detecting this disease (6). Nevertheless, these methods are not feasible for on-site diagnosis because of the absence of PCR equipment at poultry testing locations (7, 8). Therefore, developing an on-site testing solution for the prompt identification of avian pathogenic *E.coli* on chicken farms is crucial (9).

Loop-mediated isothermal amplification (LAMP), first described by Notomi et al. in 2,000, is a nucleic acid amplification method characterized by rapid amplification, operational simplicity, and high analytical specificity and sensitivity (10, 11); (Figure 1). A key advantage of LAMP is that it does not require advanced thermal cycling devices, allowing for amplification results to be easily observed and interpreted visually (12, 13). This feature makes LAMP a strong technical option for achieving efficient, cost-effective, and on-the-spot nucleic acid detection across various practical applications.

**Figure 1:**
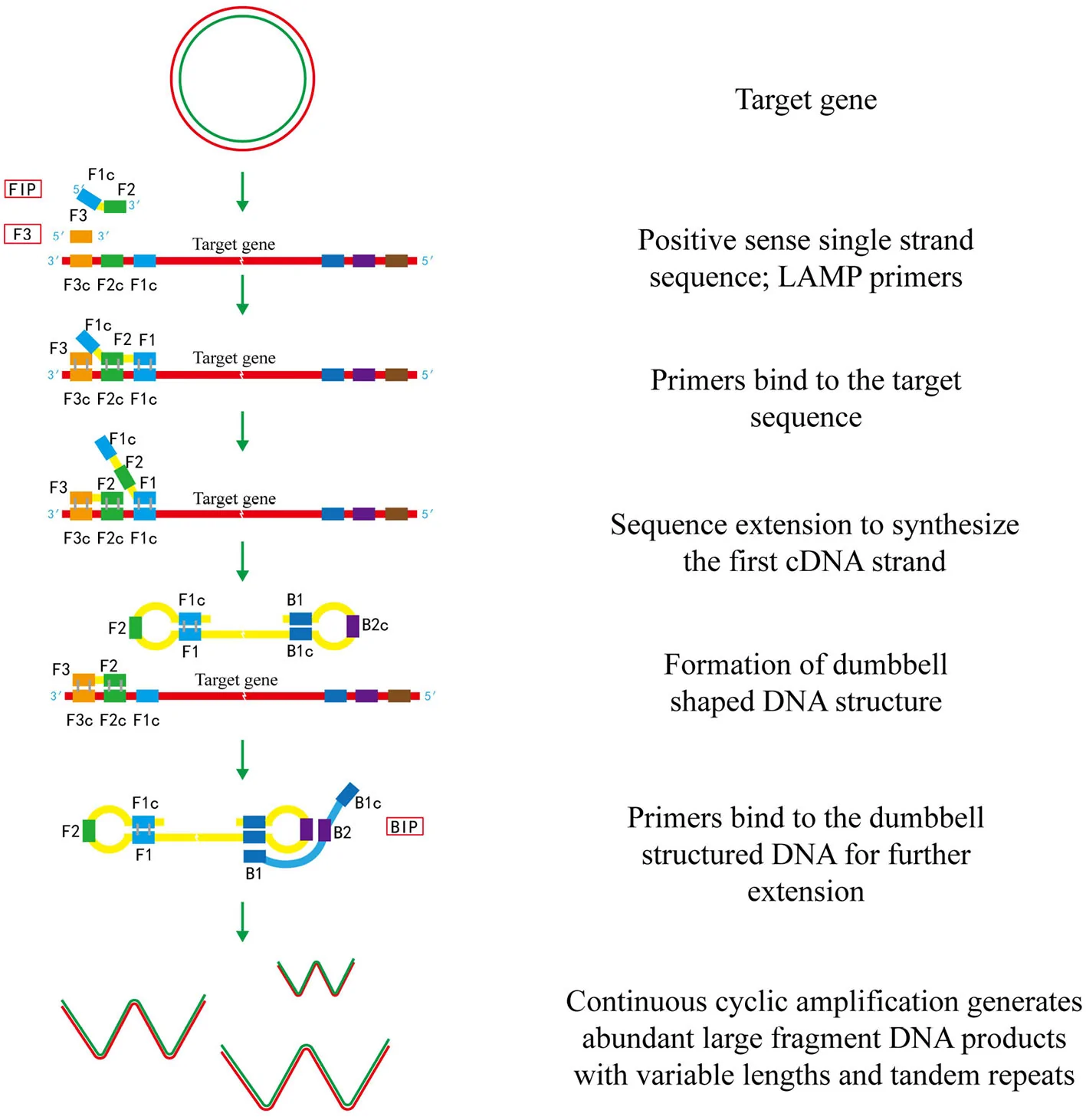
Schematic diagram of the LAMP reaction.

In the realm of avian diseases, LAMP has been widely employed for identifying various significant pathogens, such as the avian influenza virus (14), Newcastle disease virus, and avian leukosis virus (15). This method facilitates the prompt identification of pathogens present in low concentrations and supports easy on-site testing in poultry operations, providing essential technical support for effective monitoring, prevention, and management of avian diseases (16).

In this study, we developed and optimized a visual LAMP assay for rapid detection of *E. coli* in poultry-associated samples and evaluated its analytical specificity, limit of detection, agreement with bacterial culture, and performance in field samples.

## 2 Materials and methods

### 2.1 Sample collection, bacterial identification, and DNA extraction

A total of 650 samples, including anal swabs, feces, cecal contents, soil, feed, and water, were randomly collected from 11 poultry farms in Jilin Province. Among these, cecal contents were aseptically collected from 30 dead geese and chickens that had been clinically diagnosed with colibacillosis and showed pericarditis, perihepatitis, and peritonitis at necropsy. Bacterial isolates were characterized on the basis of colony morphology, culture characteristics, biochemical tests, and molecular identification targeting the 16S rRNA gene (reference sequence NR_024570) (Table 1). Genomic DNA was extracted from each isolate using the TIANamp DNA Kit (Tiangen Biotech, China) according to the manufacturer's instructions.

**Table 1:** Primer of 16S rDNA.

Gene	sequence (5^′^ → 3^′^)
16S rDNA	AGAGTTTGATCCTGGCTCAG
	GGTTACCTTGTTACGACTT

### 2.2 Design and synthesis of LAMP primers

Four LAMP primer sets were designed against highly conserved sequences identified among the *E. coli* isolates. Sequence-alignment results for representative isolates are provided in Supplementary Image 1. The candidate amplification primers, including two outer primers (F3 and B3), one forward inner primer (FIP), one backward inner primer (BIP), one forward loop primer (LF), and one backward loop primer (LB), were designed using the Primer Explorer V5 (https://primerexplorer.jp/lampv5/index.html) and synthesized by Sangon Biotechnology (Table 2).

**Table 2:** LAMP primers used in this study.

LAMP Primers	Primers name	Sequence(5^′^-3^′^)
Primer 1	F3-1	AAGCGGCTCTCTGGTCTG
	B3-1	CATGCTCCACCGCTTGTG
	FIP-1	CGGAAACCCTCCAACACCTAGC GAAAGCATGGGTAGCGAACA
	BIP-1	CAGTGCCGCAGCTAACGCAA CCCCCGTCAATTCCTTTGAG
Primer 2	F3-2	CGCAATAAGCATTCCGCCT
	B3-2	GAGCTGACGACAGCCATG
	FIP-2	ACATGCTCCACCGCTTGTGC GTACGACCGCAAGGTTGAA
	BIP-2	ACGCGAAGAACCTTACCAGGTC CCTGTCATTTTGTCCCCGAA
Primer 3	F3-3	TAGGCGACGATCCCTAGC
	B3-3	TTACTCCCTTCCTCCCCG
	FIP-3	GCTGCCTCCCGTAGGAGTCT TCTGAGAGGATGACCAGCC
	BIP-3	TATTGCACAATGGGCGCAAGC ACCCGAAGGCCTTCTTCA
Primer 4	F3-4	TCTCGTAGAGGGGGGTAGA
	B3-4	CGTTAGCTCCGGAAGCCA
	FIP-4	TCAGTCTTCGTCCAGGGGGC CAGGTGTAGCGGTGAAATGC
	BIP-4	GCTCAGGTGCGAAAGCGTGG ACCTCCAAGTCGACATCGTT

### 2.3 Optimization of LAMP primers and reaction temperature

The LAMP reaction was conducted in a total volume of 25 μL, which included 2.5 μL of 10 × ThermoPol buffer, 3.5 μL of a 10 mmol·L^−1^ dNTP Mix, 1.5 μL of 100 mmol·L^−1^ MgSO4, and 1 μL each of inner and outer primers. Furthermore, 2 μL of Bst DNA polymerase (8 U·μL^−1^), 1 μL of DNA template (The limit of detection (LOD) was 100 pg/μL), and 3 μL of sterile RNase-free water were added. To determine the optimal reaction temperature, reactions were performed at temperatures ranging from 61 to 65 °C at 1 °C intervals while all other reaction conditions were kept constant. Following incubation, 0.5 μL of SYBR Green I dye was introduced. A preliminary evaluation was carried out by observing color changes under both natural and UV light for fluorescence detection. To identify the best primer combination, 5 μL of the LAMP product was analyzed using 2% agarose gel electrophoresis.

### 2.4 Optimization of LAMP reaction system

A single-variable approach was used to evaluate the effects of reaction temperature, and Bst DNA polymerase volume. Reaction times of 15, 30, 45, 60, and 75 min were evaluated. The volume of Bst DNA polymerase was evaluated from 0.1 to 2 μL in increments of 0.3 μL. The optimal conditions were selected based on color change, fluorescence intensity, and the clarity and intensity of the electrophoretic bands produced by the amplification products.

### 2.5 SYBR green quantitative qPCR reaction

SYBR Green quantitative real-time PCR (qPCR) was performed as a comparator using primers described previously (6). Each 20-μL reaction contained 10 μL of TB Green Premix Ex Taq II (TaKaRa, Japan), 0.2 μL each of the forward and reverse primers, 1 μL of DNA template, and 8.6 μL of ultrapure water. Cycling conditions consisted of an initial denaturation at 95 °C for 30 s, followed by 40 cycles of 95 °C for 5 s and 60 °C for 30 s.

### 2.6 Evaluation of LAMP reaction specificity

The LAMP assay's specificity was assessed by utilizing DNA extracted from eight different non-target bacterial strains: *Leptospira interrogans*, (*L. interrogans*); (57620), *Staphylococcus aureus* (*S. aureus*); (26624), *Enterococcus faecalis*, (*E. faecalis*); (32482), *Listeria monocytogenes*, (*L. monocytogenes*); (54023), Salmonella typhimurium, (*S. typhimurium*); (50994); *Klebsiella pneumoniae, K. pneumoniae*) (CPCC 160022), *Proteus mirabilis* (*P. mirabilis*); (4926); S*higella flexneri*, (*S. flexneri*); (51572). These experimental strains were obtained from the China General Microbiological Culture Collection Center (CGMCC). For the non-target control, DNase-/RNase-free DEPC water was employed. Each DNA sample underwent amplification following the optimized LAMP protocol.

### 2.7 Evaluation of LAMP reaction sensitivity

To assess the sensitivity of the enhanced LAMP method, serial dilutions of *E.coli* at an initial concentration of 3.6 × 10^5^ CFU/mL were prepared in tenfold increments for sensitivity testing, with DNase-/RNase-free DEPC water serving as a non-target control (NTC). DNA was extracted from the bacterial samples individually to be used as templates, which were then amplified following the refined LAMP procedure. Amplification products were evaluated visually and by agarose-gel electrophoresis. The lowest bacterial concentration that yielded a positive result was defined as the limit of detection.

### 2.8 Comparative study on the application of three detection methods

Two hundred clinical samples were randomly selected for amplification, including a negative control (NTC). All parallel target amplifications were conducted within a standardized and unified system. Using conventional bacterial isolation and culture as the gold standard, both LAMP visual detection and conventional PCR were performed simultaneously. Following multi-batch repeated validation, the Cohen's Kappa coefficient and key ROC diagnostic parameters were statistically analyzed (17).

### 2.9 Application of the LAMP assay in isolated *E.coli* samples

In order to assess the effectiveness of the newly introduced LAMP technique, a selection of 100 samples was randomly taken from amplification. Purified *E. coli* DNA and DNase-/RNase-free DEPC-treated water served as the positive and no-template controls, respectively. Samples were tested under the optimized LAMP conditions, and results were evaluated by color change and agarose gel electrophoresis and compared with conventional PCR (18).

### 2.10 Statistical analysis

Statistical analyses were performed using SPSS version 25.0. Categorical variables are presented as *n* (%). Agreement among bacterial isolation, conventional PCR, and LAMP was evaluated using Cohen's kappa coefficient. A *P* value < 0.05 was considered statistically significant. Kappa values ≥0.75, 0.40–0.74, and < 0.40 were interpreted as good, moderate, and poor agreement, respectively. ROC curves and corresponding areas under the curve (AUCs) were calculated to characterize overall discriminatory performance.

## 3 Results

### 3.1 Bacterial isolation and identification

Following selective culture and purification, 632 *E. coli* isolates were recovered from 650 samples, corresponding to a recovery rate of 97.23% (632/650). Amplification of the 16S rRNA gene produced a distinct band of approximately 1,400 bp, consistent with the expected product size (Figure 2). Phylogenetic analysis showed that all 632 isolates shared >99% sequence similarity with *E. coli* strains JN613165.1, OR717618.1 and et al., supporting their identification as *E. coli* (Supplementary Image 1).

**Figure 2:**
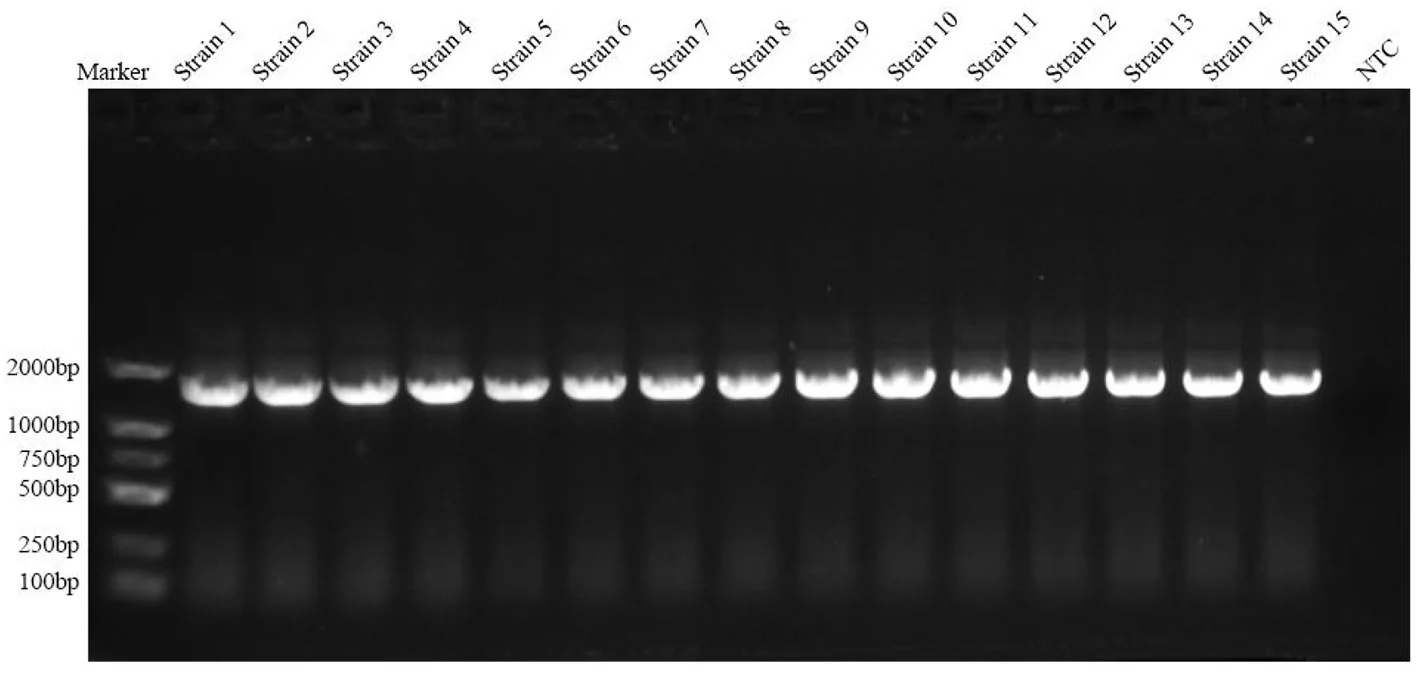
16s rDNA PCR production of isolated strains. NTC, non-target control.

### 3.2 Optimization of primes and temperature of LAMP

All four primer sets produced amplification products at temperatures ranging from 61 to 65 °C (Figure 3). Primer set 1 consistently generated the strongest and clearest bands and was therefore selected for subsequent experiments. Among the temperatures evaluated, 63 °C produced the clearest amplification pattern and was selected as the optimal reaction temperature.

**Figure 3:**
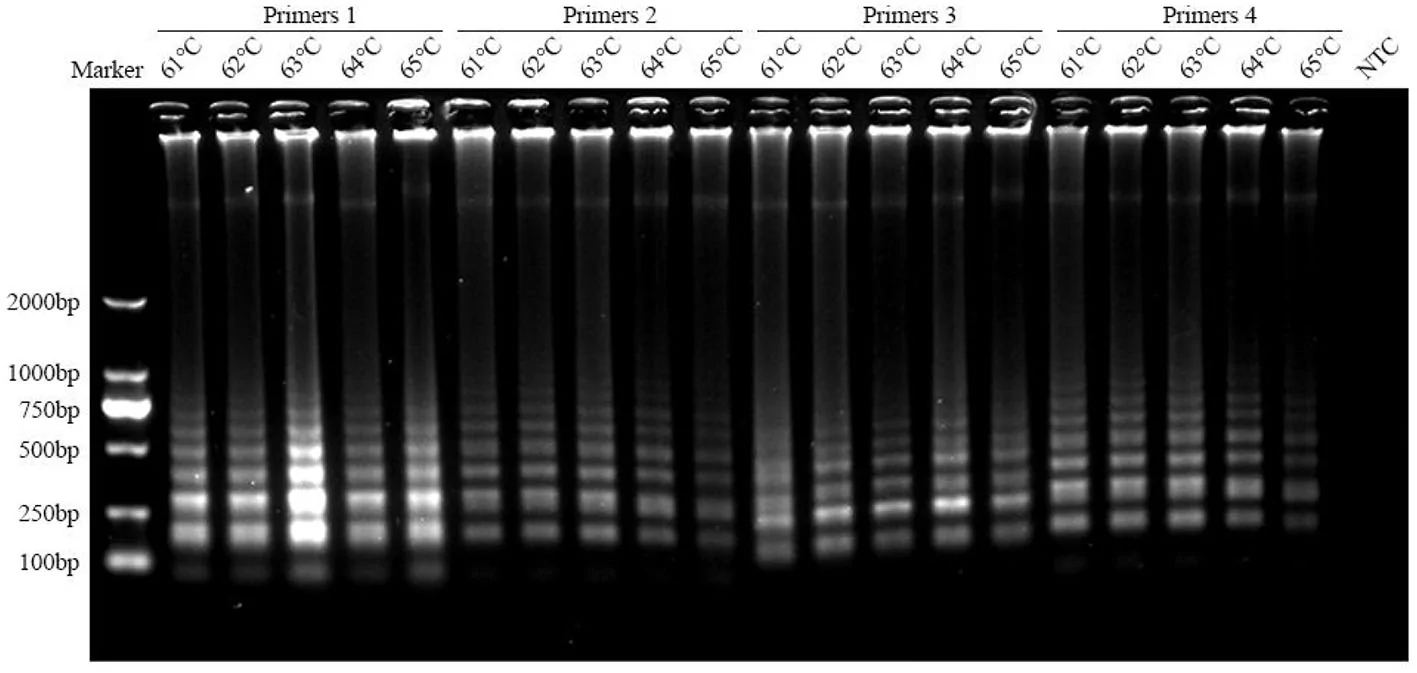
Screening of LAMP primers and annealing temperature. NTC, non-target control.

### 3.3 Optimization of reaction time

To enhance the efficiency of the LAMP reaction, experiments were performed at various time intervals. As illustrated in Figure 4A, the electrophoresis results indicated that no bands were detected at the 15 and 30-min marks. Additionally, the reaction mixture did not show any color change in regular lighting (Figure 4B), and no fluorescence was noted under UV light (Figure 4C). In contrast, at 45, 60, and 75 min, the electrophoresis revealed clear trapezoidal bands (Figure 4A), the reaction mixture displayed a yellow-green hue in normal light (Figure 4B), and pronounced fluorescence was observed under UV light (Figure 4C). Consequently, 45 min was determined to be the optimal duration for the LAMP process.

**Figure 4:**
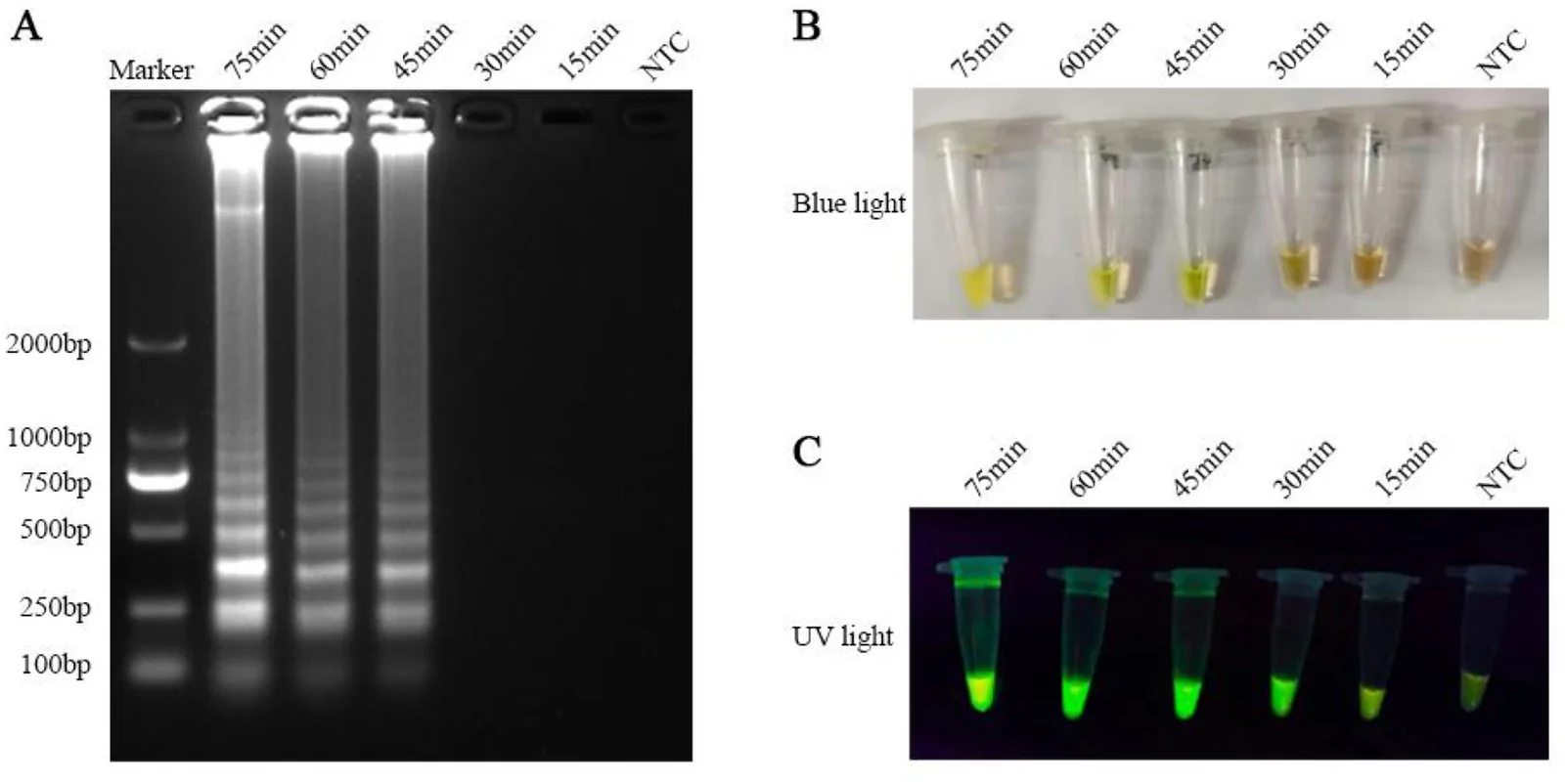
Adjusting the duration of the LAMP reaction. **(A)** Fine-tuning the LAMP reaction duration. **(B)** Modifying the LAMP reaction time using blue light. **(C)** Altering the LAMP reaction time with UV light. NTC, control for non-target.

### 3.4 Optimization of Bst DNA polymerase concentration

The LAMP procedure was performed to determine the optimal concentration of Bst DNA polymerase. When the enzyme volume was at or below 1 μL, amplification bands were not observed in the electrophoresis analysis (Figure 5A), the reaction mixture appeared orange under regular lighting (Figure 5B), and it did not show any fluorescence in normal light (Figure 5C), indicating a failure in the reaction. In contrast, when the Bst DNA polymerase concentration was 1.5 μL or higher, the electrophoresis results revealed distinct trapezoidal bands (Figure 5A), the mixture turned yellow-green in regular light (Figure 5B), and it displayed significant fluorescence when exposed to UV light (Figure 5C). Therefore, it was concluded that 1.5 μL is the optimal quantity of Bst DNA polymerase for this LAMP technique.

**Figure 5:**
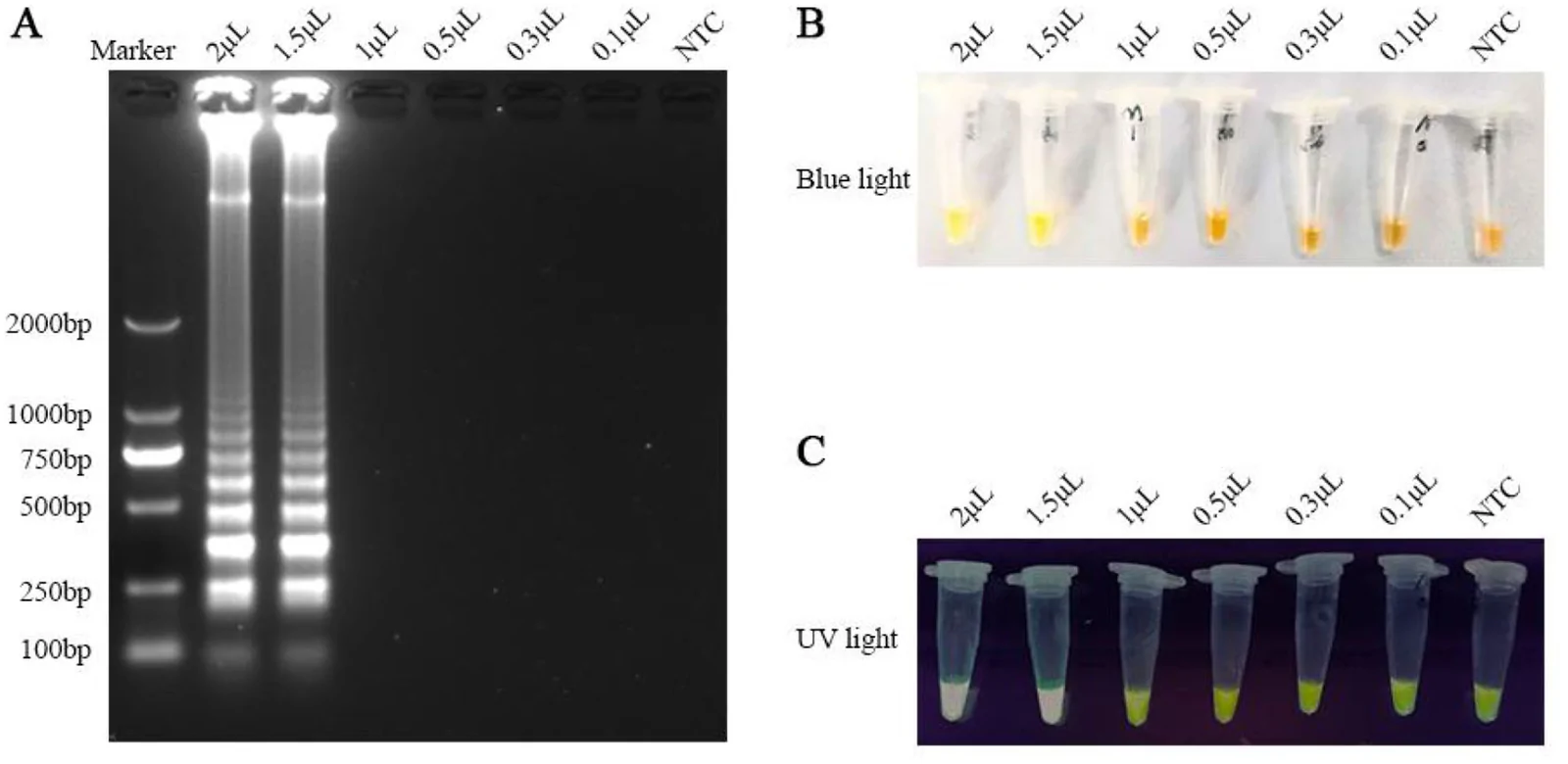
Optimization of the concentration of Bst DNA polymerase for LAMP. **(A)** Adjusting the levels of Bst DNA polymerase through PCR. **(B)** Fine-tuning the concentration of Bst DNA polymerase using blue light. **(C)** Modifying the concentration of Bst DNA polymerase with UV light. NTC, control for non-target.

### 3.5 Specificity assessment of LAMP assay

The specificity of the LAMP assay was evaluated using DNA samples obtained from eight distinct strains. As illustrated in Figure 6A, no bands were observed in the reactions involving the seven strains and the negative controls; however, distinct bands were evident in the positive *E.coli* strain (Figure 6B). Additionally, the findings indicated that only the LAMP assay for *E.coli* produced a yellow-green color under normal lighting conditions (Figure 6C) and demonstrated significant fluorescence when exposed to UV light (Figure 6D). The bacterial samples were also verified using SYBR Green qPCR, and only *E.coli* samples tested positive with cycle threshold values between 30 and 32 (Figure 6E). These outcomes suggest that the primers utilized in this study for detecting *E.coli* are specific and suitable for identifying clinical samples of *E.coli*.

**Figure 6:**
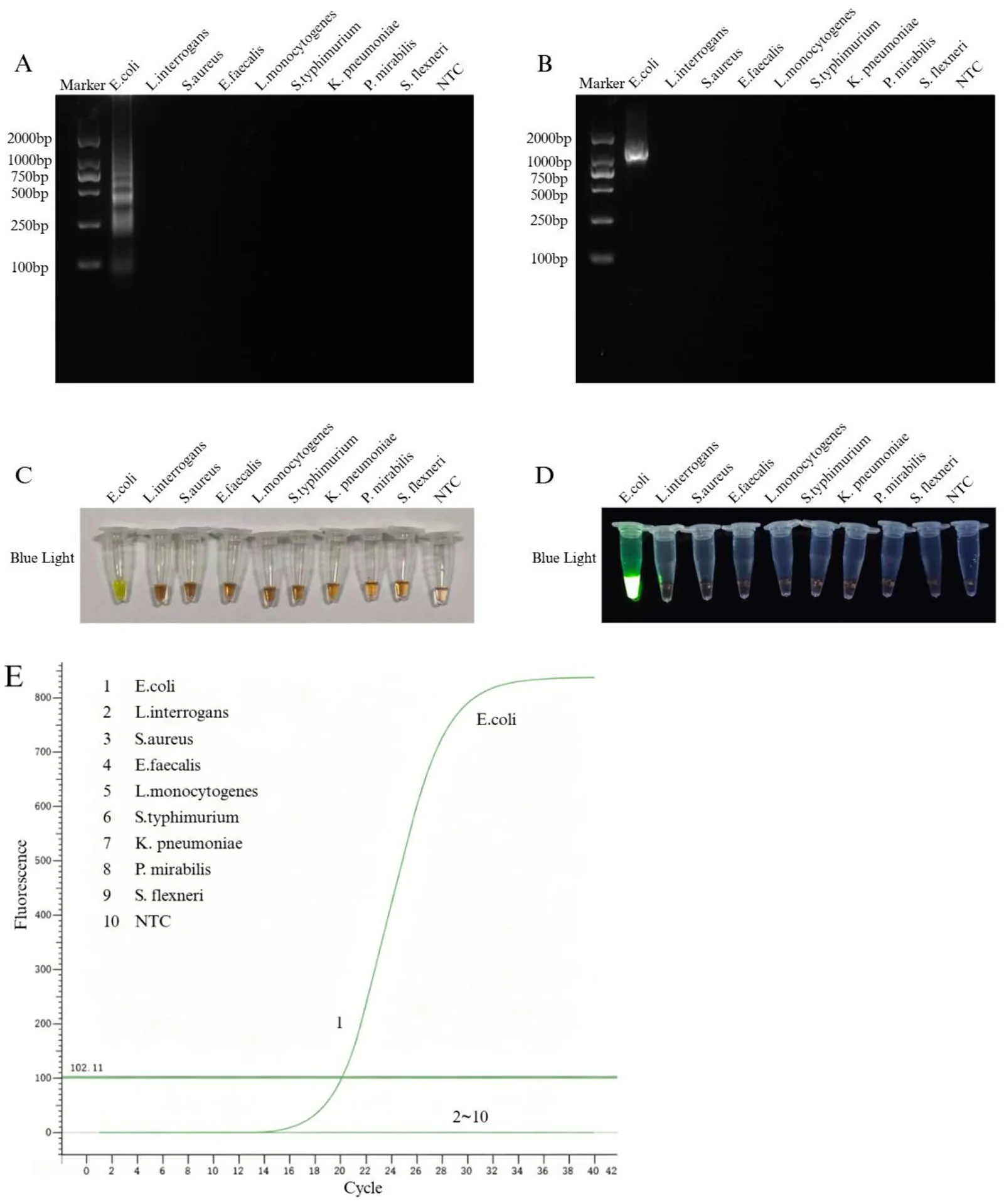
SpecifIcity evaluation of the LAMP reaction. **(A)** Assessment of the specificity of the LAMP reaction using PCR. **(B)** Assessment of the specificity of the PCR reaction. **(C)** Assessment of the specificity of the LAMP reaction utilizing blue light. **(D)** Assessment of the specificity of the LAMP reaction using UV light. **(E)** Assessment of the specificity of the qPCR. NTC, control for non-target.

### 3.6 Sensitivity assessment of LAMP assays

The LAMP assay's sensitivity for *E.coli* was determined using a 10-fold serial dilution, with concentrations ranging from 3.6 × 10^5^ to 3.6 × 10^0^ CFU/mL. At a detection level of 3.6 CFU/mL, amplification bands were visible through electrophoresis (Figure 7A), the reaction mixture exhibited a yellow-green color under normal lighting (Figure 7B), and it displayed strong fluorescence under UV light (Figure 7C), confirming that the detection threshold for LAMP was 3.6 CFU/mL. Furthermore, the sensitivity of conventional PCR and SYBR Green qPCR assays was also evaluated, revealing a detection limit of 3.6 × 10^3^ CFU/mL (Figures 7D, E). These findings suggest that the LAMP assay is 1,000 times more sensitive than the conventional PCR method for detecting *E.coli*.

**Figure 7:**
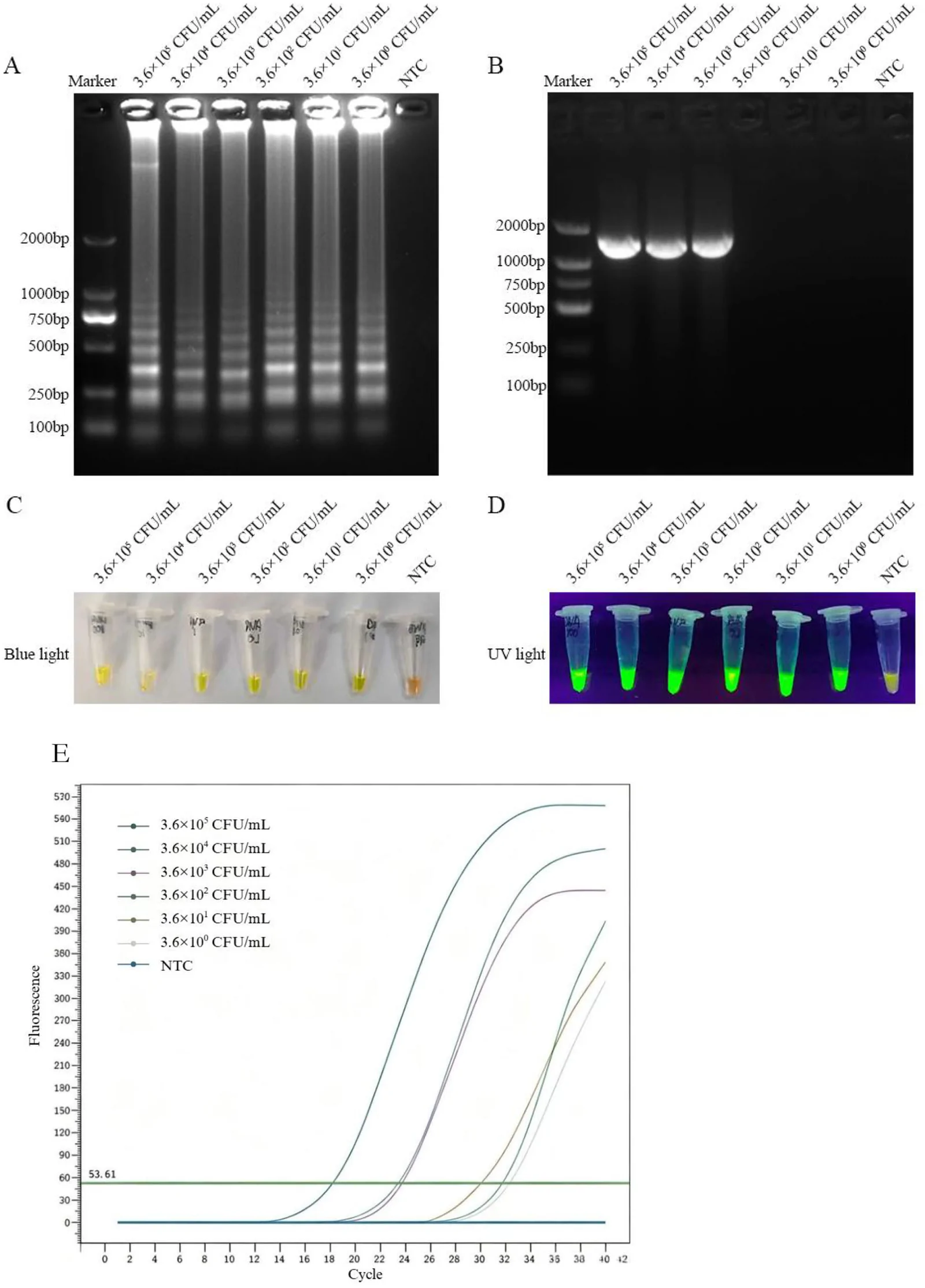
Sensitivity evaluation of the LAMP reaction. **(A)** Outcomes of LAMP amplification. **(B)** LAMP amplification outcomes for templates of different concentrations using blue light. **(C)** LAMP amplification outcomes for templates of various concentrations under UV light. **(D)** PCR amplification outcomes for templates of varying concentrations. **(E)** qPCR amplification results of different concentration templates. NTC: Control for non-target.

### 3.7 Diagnostic agreement

The diagnostic performance of the LAMP assay was compared with bacterial culture and conventional PCR, and the results are summarized in Table 3. Relative to the culture-based gold standard, the LAMP assay yielded a Cohen's kappa coefficient of 0.51, indicating substantial inter-method consistency. LAMP exhibited excellent comprehensive diagnostic performance, with 82.1% analytical sensitivity, a false positive rate of 31.8%, and an AUC value of 0.725. In comparison, conventional PCR showed a kappa value of 0.73, classified as moderate consistency with the gold standard, which was significantly lower than that of LAMP (*P* < 0.05). Moreover, conventional PCR presented a sensitivity of 100%, a false positive rate of 29.5.%, and an AUC of 0.852, with its overall diagnostic accuracy statistically inferior to the LAMP assay.

**Table 3:** Comparison of the application of the LAMP method with bacterial isolation or PCR methods.

Combination comparison	Kappa	Sensitivity	False positive	AUC	95% CI
LAMP vs. PCR	0.73	100%	29.5%	0.852	0.61–0.85
LAMP vs. Bacterial Isolation Method	0.51	82.1%	31.8%	0.752	0.778–0.926

### 3.8 Evaluation in poultry-associated samples

The LAMP assay was further evaluated using 100 samples collected from different sites (Table 4).Findings indicated that the LAMP method achieved a detection rate of 92%, with all positive samples showing complete agreement with PCR results. From these, 20 positive samples were randomly chosen for retesting using LAMP (Figure 8A), which exhibited a yellow-green hue under normal lighting conditions (Figure 8B) and pronounced fluorescence under UV light (Figure 8C) following the LAMP process, as detailed in Table 4. Results for these 20 samples were fully concordant with conventional PCR (Figure 8D and Table 4), supporting the repeatability of the assay in the specimens evaluated.

**Table 4:** Evaluation of LAMP vs. traditional PCR methods for identifying *E. coli* in a set of 100 clinical specimens.

Detection method	Sample size	Positive samples	Positivity	Positivity samples Concordance rate
PCR	100	92	92%	100%
LAMP	100	92	92%	100%

**Figure 8:**
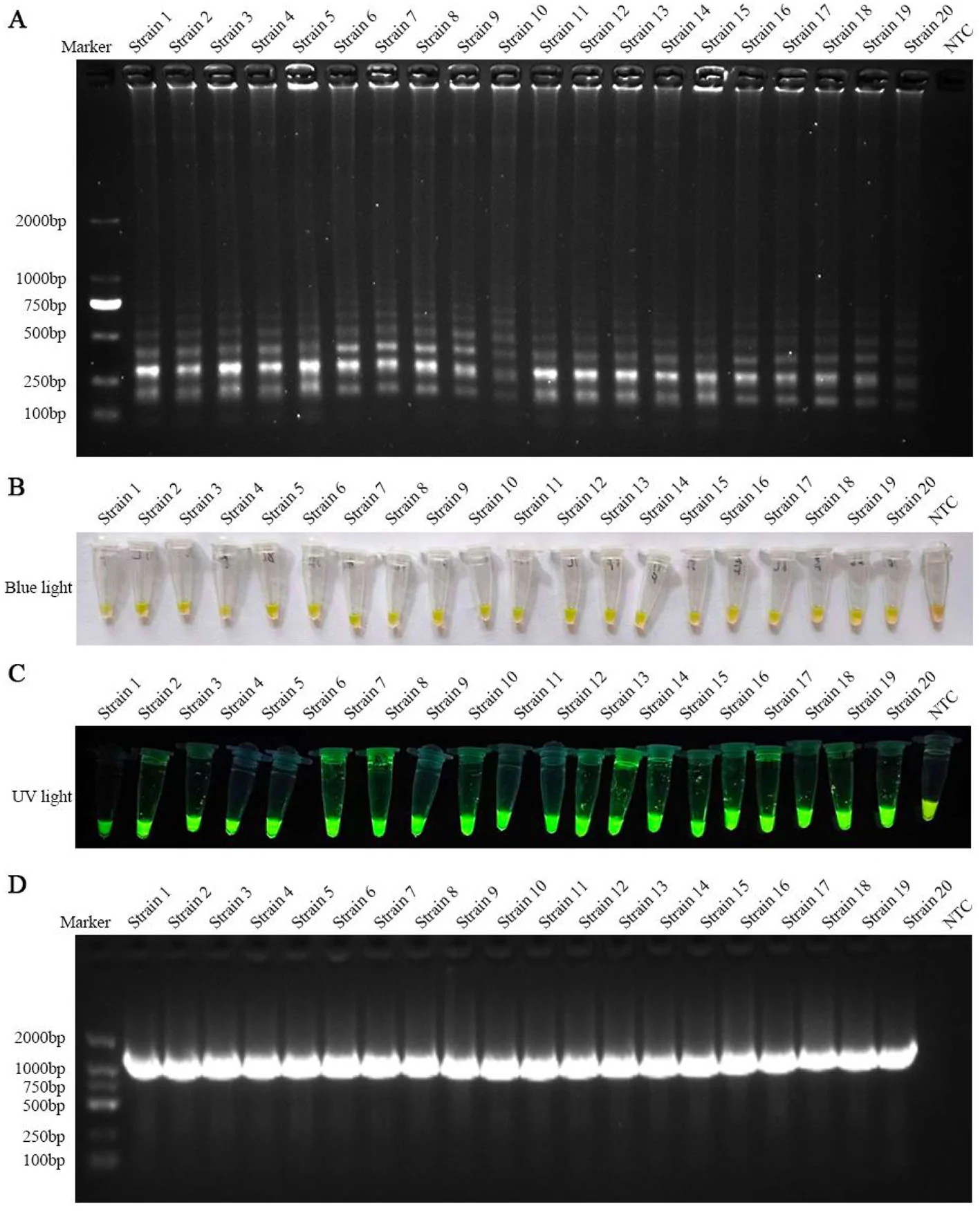
LAMP testing outcomes in clinical specimens. **(A)** Results of LAMP amplification. **(B)** LAMP amplification results observed under blue light. **(C)** LAMP amplification results observed under UV light. NTC: Control for non-target. **(D)** Outcomes of PCR amplification.

## 4 Discussion

Pathogenic strains of *Escherichia coli* are widely recognized for their ability to induce various diseases, resulting in high mortality rates among chicks and juvenile birds (19). In adult poultry, these harmful bacteria can also lead to decreased egg production, inefficient feed conversion, and hindered growth (20). Furthermore, *E. coli* contamination poses a significant risk to food safety, as infected poultry products are a major source of foodborne pathogens that can impact human health, raising considerable public health concerns (21, 22). Therefore, collecting and studying avian *E. coli* is crucial for a comprehensive understanding of its pathogenic traits, which is essential for effective clinical responses and the development of preventive strategies. In this study, 650 samples were obtained from farms across 11 regions in Jilin Province, resulting in the successful isolation of 632 *E. coli* strains, yielding an impressive isolation rate of 97.23%, surpassing the 86% noted by Kuntaman (23). Our results indicates notable variations in the prevalence of avian *E. coli* linked to different regions and farming practices, suggesting that avian colibacillosis is widespread in chicken farms throughout Jilin Province.

Rapid on-site detection can support timely management of poultry health risks associated with *E. coli*. Conventional culture and PCR require specialized equipment, trained personnel, and relatively long turnaround times (24, 25). In contrast, LAMP facilitates real-time detection, enabling poultry farmers to promptly isolate infected birds, enforce biosecurity protocols, and adjust management strategies to mitigate disease transmission (26). Furthermore, the LAMP technique requires only a water bath or heating block to maintain a stable temperature for on-site detection (27). In this study, we present a LAMP method for the rapid identification of *E. coli* in poultry operations. The LAMP assay developed here completes detection in approximately 2 h, while traditional PCR takes around 6 h. Notably, LAMP generates amplification outcomes that are immediately interpretable via direct naked-eye observation, eliminating the post-processing step required by conventional PCR: gel electrophoresis, a labor-intensive workflow that consumes over 30 min of hands-on time when accounting for both gel casting and the electrophoretic separation process. To further streamline the assay for equipment-free, point-of-use application, we implemented an optimized colorimetric readout: a pH-responsive dye is pre-added to the LAMP reaction system, and the continuous pH drop driven by robust proton release during the DNA polymerase-catalyzed isothermal strand displacement amplification triggers a highly distinguishable, readily visible color shift of the reaction mixture to yellow-green (18). LAMP technology is recognized for its exceptional sensitivity and efficiency, making it well-suited for the swift identification and control of diseases in livestock (28, 29). Its application has been extensive in the realm of poultry disease prevention and management (30, 31). Although numerous LAMP-based strategies have been successfully developed for specific detection of pathogenic *E. coli* O157 in food and water (32, 33), the application of such amplification tools in airborne bacterial monitoring is still severely underexplored. To address this unmet need, it was reported that a tailored *E. coli*-specific LAMP protocol fully integrated with a novel custom air sampling system, enabling equipment-free, streamlined on-site detection of target airborne pathogenshad (34). However, there is a lack of studies focusing on the use of LAMP for detecting avian colibacillosis in poultry. In this research, we developed a visual detection approach for *E. coli* in poultry farming using LAMP technology. The LAMP assay developed in this study enables rapid on-site detection of avian pathogenic *Escherichia coli* (APEC) within 45 min under a constant temperature of 63°C.The primers developed in this study, targeting the virulence genes, ompT, iutA and tsh based on alignment of conserved sequences from isolated avian *Escherichia coli* strains, exhibit high specificity and serve as an ideal molecular tool for the rapid identification and on-site screening of pathogenic *E. coli* in poultry-derived samples (35).This method allows for rapid identification of *E. coli*, demonstrating high selectivity, which will aid in the early diagnosis and management of avian *E. coli* infections (36, 37).

The LAMP technique demonstrates superior analytical sensitivity and detection limits compared to various other assays (38). Multiple studies have established that the detection threshold for the LAMP method ranges from 1 to 100 CFU/ml for *E. coli*, making it 10 to 100 times more sensitive than traditional PCR methods (39, 40). In research focused on *E. coli* O157, a sensitivity of 9.2 CFU/ml was noted (41). Additionally, a separate investigation into the presence of *E. coli* in water revealed that the LAMP method could detect as few as 1 CFU/ml (42). This study found that the detection limit for LAMP was 3.6 CFU/ml, significantly lower than the 3.6 x 10^3^ CFU/ml limit of conventional PCR, indicating that LAMP is more effective and accurate for identifying *E. coli* than traditional PCR and the Bacterial Isolation Method. Based on a systematic comparison and verification of the detection sensitivity of the LAMP assay, this study further randomly selected 100 clinical samples to conduct a repeatability validation experiment. At the same time, a parallel comparison was performed between the results of the LAMP assay and the PCR assay, which reconfirmed the high consistency between the LAMP method and PCR detection from the perspective of clinical samples. Despite some drawbacks, such as the unsuitability of LAMP products for cloning or sequencing due to their complex, cauliflower-like DNA structures (43, 44), the LAMP assay is preferred over conventional PCR for the swift detection of avian *colibacillosis*, owing to its heightened sensitivity and the ability to produce visually discernible results. Building on the current performance validation of the LAMP detection system, our future work will further explore and optimize the full workflow of pathogen detection through the systematic evaluation of alternative solutions for critical supporting modules, including sample collection and processing, and heating systems.

## 5 Conclusions

A colorimetric LAMP assay has been developed for the identification of avian *E. coli*. This method can accurately and repeatability detect *E. coli* concentrations as low as 3.6 CFU/mL, surpassing traditional PCR techniques and bacterial isolation. The entire procedure is completed in just 45 min at a specific temperature of 63 °C, eliminating the need for PCR machinery and complex protocols. The results of the amplification are observable without any special equipment. Therefore, the LAMP method outlined in this research has the potential to serve as an effective molecular diagnostic tool for on-site detection of *E. coli* in poultry farming.

## Data Availability

The original contributions presented in the study are publicly available. This data can be found here: NCBI SRA repository, accession number: BioProject accession number: PRJNA1528862, https://www.ncbi.nlm.nih.gov/bioproject/?term=PRJNA1528862.

## References

[1] Sani HM Iliyasu MY Alhaji SY Usman NI Abdullahi US. Virulence phenotype of avian pathogenic *Escherichia coli* (APEC) and prevalence of colibacillosis among poultry birds in bauchi metropolis, Nigeria. Int J TROPICAL DIS Health. (2025) 46:1–11. doi: 10.9734/ijtdh/2025/v46i11616

[2] Asefi N Majidzadeh-A K. Technical aspects of loop-mediated isothermal amplification (LAMP) assay in cancer. Exp Rev Mol Diagn. (2025) 25:681–94. doi: 10.1080/14737159.2025.2552820 40886110

[3] Shah SA Mir MS Kamil SA Shafi M Rather MA Khan AA . Serogroup prevalence, virulence profile and antibiotic resistance of avian pathogenic *Escherichia coli* isolated from broiler chicken. Vet Sci. (2025) 12:592. doi: 10.3390/vetsci12060592 40559829 PMC12197638

[4] Long Y Sun S Mei H Zhou D Zhou H Fang Z . RT-LAMP-CRISPR/Cas12b-based hand-pressure-actuated microfluidic chip for rapid and portable detection of severe fever with thrombocytopenia syndrome virus. Talanta. (2025) 301:129277. doi: 10.1016/j.talanta.2025.129277 41448060

[5] Guo J Shan Y Hu G Zhu X Zhao K Wu T . Rapid visual detection of Monkeypox virus by one-step LAMP-CRISPR/Cas12b assay. Virol J. (2025) 22:151. doi: 10.1186/s12985-025-02780-0 40394594 PMC12093717

[6] Zhu K Kong J Lao F Zhao L Chen A Li H. Rapid detection method for *Escherichia coli* O157:H7 and salmonella typhimurium in food based on the combination of loop-mediated isothermal amplification and hybridization chain reaction. Anal Chim Acta. (2025) 1374:344512. doi: 10.1016/j.aca.2025.344512 40983424

[7] Zhang A Lan X Zhou H Zhou T Xu Z Cheng X . A rapid and visual dual LAMP-LFD assay for on-site simultaneous detection of influenza a virus (H1N1) and respiratory syncytial virus (RSV). Front Microbiol. (2025) 16:1678396. doi: 10.3389/fmicb.2025.1678396 41292689 PMC12640905

[8] Peng LY Shi HT Gong ZX Yi PF Tang B Shen HQ . Protective effects of gut microbiota and gut microbiota-derived acetate on chicken colibacillosis induced by avian pathogenic *Escherichia coli*. Vet Microbiol. (2021) 261(prepublish):109187. doi: 10.1016/j.vetmic.2021.109187 34399296

[9] Mihailovskaya VS Selivanova PA Kuznetsova MV. Prevalence of pathogenicity island markers among commensal, avian pathogenic, and uropathogenic *Escherichia coli* strains. Bull Exp Biol Med. (2025) 179:1–6. doi: 10.1007/s10517-025-06485-w 40996648

[10] Li H Nie Y Wu Y Cao Y Liu W Zhao R . Portable microfluidic-LAMP assay for rapid on-site detection of eight highly pathogenic viruses. Anal Chimica Acta. (2025) 1365:344236. doi: 10.1016/j.aca.2025.344236 40484541

[11] Yu L Jin X Yang T Shu Q Li W Lin X. A portable digital RT-LAMP platform for rapid detection of various viruses. Sensors Actuators: B Chem. (2026) 451:139320. doi: 10.1016/j.snb.2025.139320

[12] Gallas NA Fadel ME Altammar KA Awadi Y Ben Aissa R. Pathovars, occurrence and characterization of plasmid-mediated quinolone resistance in diarrhoeal *Escherichia coli* isolated from farmers and farmed chickens in Tunisia and Nigeria. Lett Appl Microbiol. (2024) 77:ovae043. doi: 10.1093/lambio/ovae043 38653718

[13] Zhang Y Ouyang G Zheng D Chen X Mao R. Optimization of loop-mediated isothermal amplification harnessing a novel primer design targeting at short DNA sequence. Microchem J. (2026) 221:117092. doi: 10.1016/j.microc.2026.117092

[14] Glazunova A Sevskikh T Kudryashov D Sindryakova I Kolbasova O Erokhina M . Optimization of loop-mediated isothermal amplification for avian influenza detection. Animals. (2025) 15:2983. doi: 10.3390/ani15202983 41153910 PMC12560903

[15] Nair S Madhu KM. One-step RT-LAMP: a method for closed-tube colorimetric detection of RNA viruses in plants. Methods Mol Biol. (2026) 2966:67–78. doi: 10.1007/978-1-0716-4746-2_5 41028576

[16] Chantanasaro T Sararat C Yolai N Suttirat P Nawattanapaiboon K Chauvatcharin S . Modeling the effectiveness of RT-PCR, RT-LAMP, and antigen testing strategies for COVID-19 control. BMC Infect Dis. (2025) 25:1351. doi: 10.1186/s12879-025-11793-7 41107872 PMC12535133

[17] Jiddu J Madalyn J Nicolle B Li Z Pratima A Reshma R Characterization Characterization of avian pathogenic Escherichia coli isolated from broiler breeders with colibacillosis in Mississippi[J]. Poultry (2023), 2:24–39. doi: 10.3390/poultry2010004

[18] Poussard M Pant SD Huang J Scott P Ghorashi SA. Comparative evaluation of PCR and loop-mediated isothermal amplification (LAMP) assays for detecting Pasteurella multocida in poultry. N Z Vet J. (2024) 73:134–42. doi: 10.1080/00480169.2024.2417921 39448061

[19] Sun A Stejskalová P Zhen X Lu H Liu X Brodský J . Low-power microfluidic RT-LAMP system with real-time fluorescence detection for portable nucleic acid testing. Mikrochim Acta. (2025) 193:42. doi: 10.1007/s00604-025-07680-2 41428227

[20] Yang Y Yang R Luo C Wang X Ma F. Loop-mediated Isothermal Amplification (LAMP): current progress and future directions in rapid nucleic acid detection. Sensors Actuators Rep. (2026) 11:100440. doi: 10.1016/j.snr.2026.100440

[21] Polpitiya K Hewadikaram M. Loop-mediated isothermal amplification (LAMP): a promising tool for rapid, point-of-care diagnosis of parasitic diseases in low-income countries. Mol Biochem Parasitol. (2025) 264:111704. doi: 10.1016/j.molbiopara.2025.111704 41077252

[22] Chen F Mao S Zhang R Yang X Huang J Kang Y . Lateral flow biosensor-assisted loop-mediated isothermal amplification assay for ultrafast, sensitive detection of African swine fever virus. Talanta Open. (2025) 12:100581. doi: 10.1016/j.talo.2025.100581

[23] Mo Y Zhu J Huang Y Hu J Su Z Zhang C. Inner primer blockers inhibit non-specific amplification in LAMP. Analyt Chimica Acta. (2026) 1385:345020. doi: 10.1016/j.aca.2025.345020 41526128

[24] Shen Y Zheng Y Li M Du Y Yang H Li F . A hydroxynaphthol blue-based loop-mediated isothermal amplification assay for closed-tube detection of the streptomycin resistance gene aadA1 in salmonella. Vet Sci. (2025) 12:1094. doi: 10.3390/vetsci12111094 41295732 PMC12656972

[25] Zhang X Zhao Y Zeng Y Zhang C. Evolution of the probe-based loop-mediated isothermal amplification (LAMP) assays in pathogen detection. Diagnostics (Basel). (2023) 13:1530. doi: 10.3390/diagnostics13091530 37174922 PMC10177487

[26] Segundo F Kwame O Ana P Frederick KJ. Evaluation of an ultra-portable pocket-sized device for running Loop mediated isothermal amplification (LAMP) assays for rapid detection of sweetpotato viruses. Gates Open Res. (2025) 9:91. doi: 10.12688/gatesopenres.16369.1 41141835 PMC12552739

[27] Skenndri S Lahkak FE Kamli TE Agargar Z Maane IA Nassik S. A dual-gene colorimetric LAMP assay for genus-level detection of salmonella and specific identification of the non-motile serovar S. Gallinarum Gallinarum. Int J Mol Sci. (2025) 26:12083. doi: 10.3390/ijms262412083 41465510 PMC12732879

[28] Li W Song XX Ru SS Hao J Cao CY Zhang X. Development of PCR, qPCR and LAMP methods for the detection of *Spirometra mansoni* (Cestoda: Diphyllobothriidae) in the faeces of dogs and cats. Food Waterborne Parasitol. (2025) 41:e00291-e. doi: 10.1016/j.fawpar.2025.e00291 41079405 PMC12508883

[29] Matić S Cognolato L Sanna M Mezzalama M Laurenti R Spadaro D. Development of lab-on-a-chip LAMP and real-time PCR assays to detect *aflatoxigenic aspergillus* flavus and *aspergillus parasiticus* in hazelnuts. Toxins. (2025) 17:510. doi: 10.3390/toxins17100510 41150210 PMC12568288

[30] Song BS Baek YH Kim EH Kwon HI Kim AH Lee SH . Development of an RT-LAMP-CRISPR/Cas12a assay for rapid and specific detection of *Bandavirus dabieense*. J Microbiol. (2025) 63:e2506013. doi: 10.71150/jm.2506013 41309229 PMC13577080

[31] Castro JC Peralta FO León DF Meza EC Gavilán RG Quino W. Development and evaluation of loop-mediated isothermal amplification (LAMP) for rapid detection of *campylobacter jejuni*. Rev Peru Med Exp Salud Publica. (2025) 42:263–70. doi: 10.17843/rpmesp.2025.423.14501 41557543 PMC12679974

[32] Davidson JL Maruthumuthu MK Kamel M Mohan S Pascual-Garrigos A Dextre A . Detection of five viruses commonly implicated with Bovine Respiratory Disease using loop-mediated isothermal amplification. bioRxiv : the preprint server for biology. (2025). doi: 10.1101/2025.11.06.687045 41521899 PMC12798664

[33] Xue X He M Wang P Zhang Y. Current status of detection strategies for pathogenic *Escherichia coli*: a brief review and summary. Analyt Sci. (2025) 41:1–10. doi: 10.1007/s44211-025-00836-3 40839245

[34] Eraslan-Gültekin H Ergenc AF. Detection of *Escherichia coli* using loop-mediated isothermal amplification for a novel air sampling system. Aerosol Air Qual Res. (2025) 25:8. doi: 10.1007/s44408-025-00010-4

[35] Johnson TJ Wannemuehler Y Doetkott C Johnson SJ Rosenberger SC Nolan LK. Identification of minimal predictors of avian pathogenic *Escherichia coli* virulence for use as a rapid diagnostic tool.[J]. J Clin microbiol. (2008) 46:3987–96. doi: 10.1128/JCM.00816-08 18842938 PMC2593276

[36] Lopez MR Monteiro R Pizza M Desvaux M Rosini R. Correction: intestinal pathogenic *Escherichia coli*: insights for vaccine development. Front Microbiol. (2025) 16:1679236. doi: 10.3389/fmicb.2025.1679236 40904685 PMC12404034

[37] Saberi A Mosleh N Shomali T Naziri Z A A comparative study on two infection models of colibacillosis in broilers: clinical features pathogenesis and response to therapy. Avian Dis. (2023) 67:261–8. doi: 10.1637/aviandiseases-D-23-00032 39126413

[38] Yang N Zhang H Han X Liu Z Lu Y. Advancements and applications of loop-mediated isothermal amplification technology: a comprehensive overview. Front Microbiol. (2024) 15:1406632. doi: 10.3389/fmicb.2024.1406632 39091309 PMC11292733

[39] Tian T Bao Y Hui J Gao Y Wang L Zhao J . Analysis of avian pathogenic *Escherichia coli* (APEC) and its antimicrobial resistance risk characteristics and critical control points in laying hens. Poult Sci. (2025) 104:106049. doi: 10.1016/j.psj.2025.106049 41237586 PMC12663712

[40] Sharma AS Lee NY. Advancements in visualizing loop-mediated isothermal amplification (LAMP) reactions: a comprehensive review of colorimetric and fluorometric detection strategies for precise diagnosis of infectious diseases. Coordination Chem Rev. (2024) 509:215769. doi: 10.1016/j.ccr.2024.215769

[41] Pavon RDN Rivera WL. Loop-mediated isothermal amplification assay for visual detection of salmonella enterica serovar typhimurium in food animal meat products. Foods. (2025) 14:1731. doi: 10.3390/foods14101731 40428511 PMC12111752

[42] Kuntaman K Masfufatun M Wibisono FJ Sarassari R Setyorini W Tania POA . Assessment of the environmental transmission dynamics of *Escherichia coli*-producing ESBL in wet market broiler chickens: a high-burden food safety concern. Open Vet J. (2025) 15:2230–7. doi: 10.5455/OVJ.2025.v15.i5.40 40557073 PMC12184475

[43] Chaudhary D Poudel S Hsu CY Jia L Sukumaran AT Zhang X . Evaluation of different DNA extraction methods for the detection of clostridium perfringens by loop-mediated isothermal amplification (LAMP) assay. J Appl Poult Res. (2025) 34:100600. doi: 10.1016/j.japr.2025.100600

[44] Chaney WE Baxter J Costa MD Kuttappan VA Oelschlager M Jones M . Applied research note: effect of a postbiotic and phytogenic blend on the severity of avian pathogenic *Escherichia coli* (APEC) serotype O78 infection in a 10 week layer pullet challenge model. J Appl Poult Res. (2025) 34:100630. doi: 10.1016/j.japr.2025.100630

