# Supplementary material for: Establish a visual loop-mediated isothermal amplification (LAMP) method for rapid detection avian colibacillosis
Source: Front Vet Sci. 2026 Sep 25;13:1922461. doi: 10.3389/fvets.2026.1922461 (PMC13651099; doi:10.3389/fvets.2026.1922461)
Supplement: Supplementary file 1 [file Supplementary_file_1.pdf]

Supplementary Material

1 Supplementary Figure

1.1 Supplementary Figure

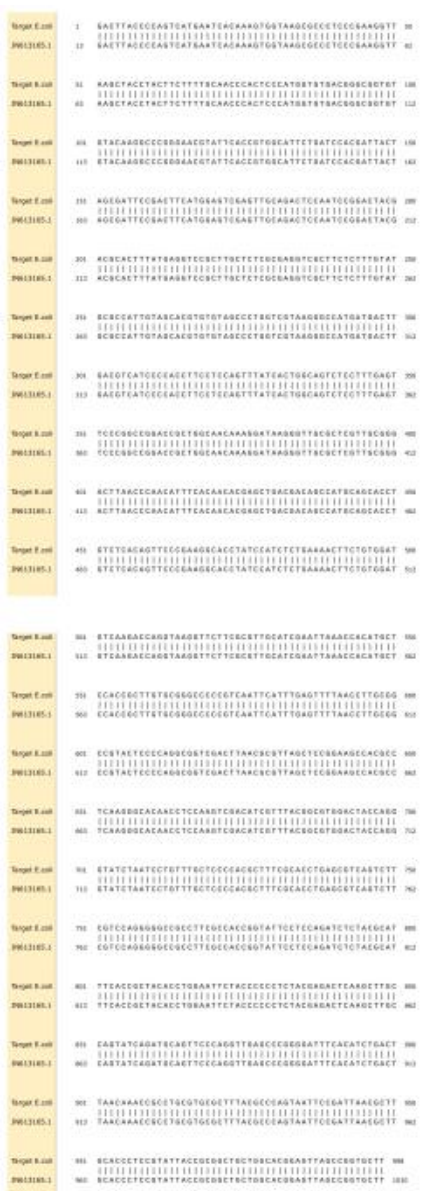

Supplementary Figure 1. Results of sequence and alignment for partial strains

(Note:>1:Contig1

TCGCTGACGAGTGGCGGACGGGTGAGTAATGTCTGGGAAACTGCCTGATGGAGGGGGA TAACTACTGGAAACGGTAGCTAATACCGCATAACGTCGCAAG

ACCAAAGAGGGGGACCTTCGGGCCTCTTGCCATCGGATGTGCCCAGATGGGATTAGCTT GTTGGTGGGGTAACGGCTCACCAAGGCGACGATCCCTAGCT

GGTCTGAGAGGATGACCAGCCACACTGGAACTGAGACACGGTCCAGACTCCTACGGGA GGCAGCAGTGGGGAATATTGCACAATGGGCGCAAGCCTGATG

CAGCCATGCCGCGTGTATGAAGAAGGCCTTCGGGTTGTAAAGTACTTTCAGCGGGGAGG AAGGGAGTAAAGTTAATACCTTTGCTCATTGACGTTACCCG

CAGAAGAAGCACCGGCTAACTCCGTGCCAGCAGCCGCGGTAATACGGAGGGTGCAAGC
GTTAATCGGAATTACTGGGCGTAAAGCGCACGCAGGCGGTTT

GTTAAGTCAGATGTGAAATCCCCGGGCTCAACCTGGGAACTGCATCTGATACTGGCAAG
CTTGAGTCTCGTAGAGGGGGGTAGAATTCCAGGTGTAGCGG

TGAAATGCGTAGAGATCTGGAGGAATACCGGTGGCGAAGGCGGCCCCTGGACGAAGAC
TGACGCTCAGGTGCGAAAGCGTGGGGAGCAAACAGGATTAGA

TACCCTGGTAGTCCACGCCGTAAACGATGTCGACTTGGAGGTTGTGCCCTTGAGGCGTG
GCTTCCGGAGCTAACGCGTTAAGTCGACCGCCTGGGGAGTA

CGGCCGCAAGGTTAAAACTCAAATGAATTGACGGGGGCCCGCACAAGCGGTGGAGCAT
GTGGTTTAATTCGATGCAACGCGAAGAACCTTACCTGGTCTT

GACATCCACGGAAGTTTTCAGAGATGAGAAGGTGCCTTCGGGAACCGTGAGACAGGTGC
TGCATGGCTGTCGTCAGCTCGTGTTGTGAAATGTTGGGTTA

AGTCCCGCAACGAGCGCAACCCTTATCCTTTGTTGCCAGCGGTCCGGCCGGGAACTCAA
AGGAGACTGCCAGTGATAAACTGGAGGAAGGTGGGGATGAC

GTCAAGTCATCATGGCCCTTACGACCAGGGCTACACACGTGCTACAATGGCGCATACAA
AGAGAAGCGACCTCGCGAGAGCAAGCGGACCTCATAAAGTG

CGTCGTAGTCCGGATTGGAGTCTGCAACTCGACTCCATGAAGTCGGAATCGCTAGTAAT
CGTGGATCAGAATGCCACGGTGAATACGTTCCCGGGCCTTG

TACACACCGCCCGTCACACCATGGGAGTGGGTTGCAAAAGAAGTAGGTAGCTTAACCTT
C

>2:Contig1

TCGCTGACGAGTGGCGGACGGGTGAGTAATGTCTGGGAAACTGCCTGATGGAGGGGGA
TAACTACTGGAAACGGTAGCTAATACCGCATAACGTCGCAAG

ACCAAAGAGGGGGACCTTCGGGCCTCTTGCCATCGGATGTGCCCAGATGGGATTAGCTT
GTTGGTGGGGTAACGGCTCACCAAGGCGACGATCCCTAGCT

GGTCTGAGAGGATGACCAGCCACACTGGAACTGAGACACGGTCCAGACTCCTACGGGA
GGCAGCAGTGGGGAATATTGCACAATGGGCGCAAGCCTGATG

CAGCCATGCCGCGTGTATGAAGAAGGCCTTCGGGTTGTAAAGTACTTTCAGCGGGGAGG
AAGGGAGTAAAGTTAATACCTTTGCTCATTGACGTTACCCG

CAGAAGAAGCACCGGCTAACTCCGTGCCAGCAGCCGCGGTAATACGGAGGGTGCAAGC
GTTAATCGGAATTACTGGGCGTAAAGCGCACGCAGGCGGTTT

GTTAAGTCAGATGTGAAATCCCCGGGCTCAACCTGGGAACTGCATCTGATACTGGCAAG CTTGAGTCTCGTAGAGGGGGGTAGAATTCCAGGTGTAGCGG

TGAAATGCGTAGAGATCTGGAGGAATACCGGTGGCGAAGGCGGCCCCCTGGACGAAGA CTGACGCTCAGGTGCGAAAGCGTGGGGAGCAAACAGGATTAG

ATACCCTGGTAGTCCACGCCGTAAACGATGTCGACTTGGAGGTTGTGCCCTTGAGGCGT GGCTTCCGGAGCTAACGCGTTAAGTCGACCGCCTGGGGAGT

ACGGCCGCAAGGTTAAAACTCAAATGAATTGACGGGGGCCCGCACAAGCGGTGGAGCA TGTGGTTTAATTCGATGCAACGCGAAGAACCTTACCTGGTCT

TGACATCCACAGAAGTTTTCAGAGATGAAAATGTGCCTTCGGGAACCGTGAGACAGGTG CTGCATGGCTGTCGTCAGCTCGTGTTGTGAAATGTTGGGTT

AAGTCCCGCAACGAGCGCAACCCTTATCCTTTGTTGCCAGCGGTCCGGCCGGGAACTCA AAGGAGACTGCCAGTGATAAACTGGAGGAAGGTGGGGATGA

CGTCAAGTCATCATGGCCCTTACGACCAGGGCTACACACGTGCTACAATGGCGCATACA AAGAGAAGCGACCTCGCGAGAGCAAGCGGACCTCATAAAGT

GCGTCGTAGTCCGGATTGGAGTCTGCAACTCGACTCCATGAAGTCGGAATCGCTAGTAA TCGTGGATCAGAATGCCACGGTGAATACGTTCCCGGGCCTT

GTACACACCGCCCGTCACACCATGGGAGTGGGTTGCAAAAGAAGTAGGTAGCTTAACCT

>3:Contig1

TCGCTGACGAGTGGCGGACGGGTGAGTAATGTCTGGGAAACTGCCTGATGGAGGGGGA TAACTACTGGAAACGGTAGCTAATACCGCATAACGTCGCAAG

ACCAAAGAGGGGGACCTTCGGGCCTCTTGCCATCGGATGTGCCCAGATGGGATTAGCTT GTTGGTGGGGTAACGGCTCACCAAGGCGACGATCCCTAGCT

GGTCTGAGAGGATGACCAGCCACACTGGAACTGAGACACGGTCCAGACTCCTACGGGA GGCAGCAGTGGGGAATATTGCACAATGGGCGCAAGCCTGATG

CAGCCATGCCGCGTGTATGAAGAAGGCCTTCGGGTTGTAAAGTACTTTCAGCGGGGAGG AAGGGAGTAAAGTTAATATCTTTGCTCATTGACGTTACCCG

CAGAAGAAGCACCGGCTAACTCCGTGCCAGCAGCCGCGGTAATACGGAGGGTGCAAGC GTTAATCGGAATTACTGGGCGTAAAGCGCACGCAGGCGGTTT

GTTAAGTCAGATGTGAAATCCCCGGGCTCAACCTGGGAACTGCATCTGATACTGGCAAG CTTGAGTCTCGTAGAGGGGGGTAGAATTCCAGGTGTAGCGG

TGAAATGCGTAGAGATCTGGAGGAATACCGGTGGCGAAGGCGGCCCCCTGGACGAAGAC TGACGCTCAGGTGCGAAAGCGTGGGGAGCAAACAGGATTAGA

TACCCTGGTAGTCCACGCCGTAAACGATGTCGACTTGGAGGTTGTGCCCTTGAGGCGTG
GCTTCCGGAGCTAACGCGTTAAGTCGACCGCCTGGGGAGTA

CGGCCGCAAGGTTAAAACTCAAATGAATTGACGGGGGCCCGCACAAGCGGTGGAGCAT
GTGGTTTAATTCGATGCAACGCGAAGAACCTTACCTGGTCTT

GACATCCACGGAAGTTTTCAGAGATGGATTGGTGCCTTCGGGAACCGTGAGACAGGTGC
TGCATGGCTGTCGTCAGCTCGTGTTGTGAAATGTTGGGTTA

AGTCCCGCAACGAGCGCAACCCTTATCCTTTGTTGCCAGCGGTCCGGCCGGGAACTCAA
AGGAGACTGCCAGTGATAAACTGGAGGAAGGTGGGGATGAC

GTCAAGTCATCATGGCCCTTACGACCAGGGCTACACACGTGCTACAATGGCGCATACAA
AGAGAAGCGACCTCGCGAGAGCAAGCGGACCTCATAAAGTG

CGTCGTAGTCCGGATTGGAGTCTGCAACTCGACTCCATGAAGTCGGAATCGCTAGTAAT
CGTGGATCAGAATGCCACGGTGAATACGTTCCCGGGCCTTG

TACACACCGCCCGTCACACCATGGGAGTGGGTTGCAAAAGAAGTAGGTAGCTTAAC)
